# Push out bond strength of resin -based endodontic sealer loaded with silver gold nanoparticles in vitro study

**DOI:** 10.1038/s41405-025-00331-x

**Published:** 2025-05-08

**Authors:** Nermine Hassan, Mona Riad, Shereen Hafez Ibrahim, Bassam Ahmed Abulnoor, Reham Hassan

**Affiliations:** 1 Lecturer of Endodontics, Faculty of Dentistry, Cairo University, Giza, Egypt; 2 Professor of Conservative Dentistry, Faculty of Dentistry, Cairo University, Giza, Egypt; 3 PhD candidate, Faculty of Dentistry, Ain Shams University, Giza, Egypt; 4 Professor of Endodontics, Faculty of Dentistry, Minia University, Minia, Egypt; 5 Professor of Endodontics, Faculty of Dentistry, Egyptian Russian University, Badr city, Egypt

**Keywords:** Root canal treatment, Gutta percha

## Abstract

**Objectives:**

The push-out bond strength to the intraradicular dentin was evaluated in this in vitro study using an epoxy-based endodontic sealer loaded with silver-gold nanoparticles. Additionally, the benefits of using the nanoparticles as a canal pretreatment before applying the sealer were evaluated in comparison to using the sealer alone.

**Methodology:**

After being decoronated and instrumented, thirty extracted human central incisors were obturated and then divided horizontally into three parts (coronal, middle, and apical) with a thickness of 2 mm. Three groups of slices were created at random: Groups I (control) and II received AH Plus sealer, AH Plus sealer loaded with silver gold nanoparticles, and III received nanoparticles as a pretreatment for the canal prior to gutta-percha and AH Plus sealer for obturation. The specimens were loaded at a rate of 0.5 mm/min utilizing a Universal Test Machine and put through a push-out test. Statistics were applied to each data collection. Additionally, the significance level was set at *P* ≤ 0.05.

**Results:**

A push-out test showed that the groups differed significantly from one another. The tested groups differed significantly in both the coronal and middle portions, with group (II) exhibiting the highest push-out strength values. Group (II) had the highest push-out strength values in the apical portion (3.66 ± 0.72), followed by group (III) (2.90 ± 1.45), while group (I) had the lowest value (2.88 ± 1.73). There was no significant difference between the groups.

**Conclusions:**

the experimental AH Plus sealer combined with silver-gold nanoparticles demonstrated the most effective adhesion followed by nanoparticles as a canal pretreatment prior to sealer application when compared to AH Plus sealer alone.

## Introduction

The purpose of root canal obturation is to produce an appropriate seal between the dentinal walls and the endodontic filling materials in addition to providing good antibacterial properties to fight the resistant microorganisms. Thus, the endodontic obturation must provide a hermetic seal and bonding to root dentin to work as a barrier to enclose any residual germs and stop infection or reinfection of the root canal system [1]. Bacterial leaking is one of the main reasons why endodontic treatments fail. A 1% shrinking of the root canal sealer has been demonstrated to provide an opening for bacteria to enter at the interface between the sealer and gutta percha or dentin [2, 3]. The sealer's job is to adhere or cement the core obturation material into the root canal and fill in any accessory or lateral canals. A hermetic seal of the root canal should be achieved by the sealer connecting to both dentin and solid filling material, ideally with dimensional stability [2]. Effective root canal therapy requires the removal or reduction of intra-canal bacteria, full root canal closure, and a high degree of filling material adaptation to the cleaned root canal area, dentin walls, and, if feasible, penetration into dentinal tubules [1]. Because of its remarkable physicochemical and biological properties, AH Plus (Dentsply, De Trey GmbH, Konstanz, Germany), an epoxy resin-based sealer, is one of the most commonly and clinically recommended endodontic sealers. Other sealers, such as bioceramic-based sealers, are also gaining popularity due to their unique properties and may be considered alternatives [4–6].

Furthermore, most root canal sealants significantly lose their antibacterial properties after setting. Compared to anaerobes, gram-positive facultative species are more resistant to the antimicrobial medications employed in endodontic therapy. *Enterococcus faecalis* (*E. faecalis*) is the most common bacterial species responsible for persistent or secondary infections, which are the main cause of endodontic treatment failure [3, 4]. Even with the use of irrigation schedules, intracanal drugs, and other kinds of equipment, microbes can thrive in the intricate root canal design [7–11]. As a result, sealers that contain antibacterially active ingredients might function better by coming into closer touch with the bacteria that remain in the root canal system.

One of the most promising areas for creating new applications in the modern world is nanotechnology. The most well-known kind of nanoproduct is nanosilver. Silver nanoparticles have been employed in many studies as an intracanal medication and as a root canal disinfectant due to their potent antibacterial activity [7, 11–13]. In recent years, numerous studies around the world have demonstrated that silver nanoparticles (Ag NPs) have the strongest antibacterial effect when compared to other antibacterial nanomaterials [12–14]. Gold nanoparticles have recently been the focus of numerous ongoing investigations because of their optical characteristics and applications in biomedicine. Because nanoparticles must first be synthesized and characterized to verify that they are the proper size, shape, and type before they can be loaded into a material, loading endodontic intracanal medicine or irrigating solution with nanoparticles is challenging or impossible. The need for sophisticated equipment and the need to constantly modify the chosen concentration using mathematical formulas limit this utilization.

A recent disinfectant made especially for dental use was launched. The product description and detailed specs have been supplied by the manufacturer. Silver nanoparticles and a trace amount of gold are present in Dental Nanotechnology's Nano Care Plus Silver Gold® (Nano Care), which is manufactured in Katowice, Poland. It was claimed by the manufacturer that the pharmacological combination Nano Care has persistent bacteriostatic action. It keeps germs from recolonizing inside the root canal system and serves as an extra cleaner of organic wastes. Silver and gold nanoparticles are guaranteed to exhibit antibacterial properties against a broad spectrum of bacterial species due to their diverse sizes, shapes, and surface energies [12]. The most common type of NPs in the Nano Care solution were AgNPs, which accounted for 99% of the particles and had an average size of 29.07 nm. Gold nanoparticles (AuNPs), which made up the remaining 1% of the particles, were suspended in 70% isopropyl alcohol and had an average size of 136.7 /nm. It is made up of many circular, discoid, spherical nanoparticles that are 48 nm in size. Due to the fact that there is only one point of interaction, the spherical form of the nanoparticles reduces the likelihood of agglomeration. Additionally, the metal nanoparticles are dispersed in a liquid carrier, such as isopropanol, which offers an additional benefit by reducing NP agglomeration through dissolution in a liquid carrier, such as methanol and isopropanol [15, 16].

According to research, the bond strength and sealer penetration may be impacted by the type of sealer applied and the root canal walls' pretreatment [3]. According to a review of the literature, no research has been done on the efficacy of NanoCare as a pretreatment for dentine in root canals or as an ingredient in sealers. The primary use of NanoCare is as a disinfectant and nanoparticle-based solution to enhance the antibacterial effects. The research question was “could it enhance/reduce bond strength? In order to determine whether using these nanobiomaterials as a root canal dentine pretreatment or loaded into the sealer could be a practical method to improve sealer bonding, this study has investigated whether combining gold and silver, particularly in a single compound, could result in a synergistic effect between their respective qualities. The null hypothesis tested that there was no significance difference among the tested groups.

## Materials and Methods

### Sample size calculation

In order to have sufficient power to execute a statistical test, a power analysis was created. Based on the findings of a previous study [17], the anticipated sample size (n) was determined to be (30) samples, or 10 samples per group, using an alpha (α) level of 0.05, a beta (β) level of 0.2 (i.e., power = 80%), and an effect size (f) of 0.5. G*Power version 3.1.9.7 was used to calculate the sample size [18].

### Study design

According to the Helsinki Declaration and its subsequent revisions, the study protocol was authorized by the Faculty of Dentistry's ethics committee at Cairo University in Egypt (54/7/24) and all participants provided their informed consent for the use of the extraxted teeth. The study design was experimental laboratory study including three groups.

### Sample selection

Thirty human maxillary central incisors were extracted for periodontal purposes. In order to verify the canal's architecture and rule out patients with caries, resorption, fractures, calcified root canals, and prior endodontic therapy, pre-operative radiographs were taken from the buccolingual and mesiodistal directions. Tooth with cavities, fissures, fractures on the root surfaces, immature root apices, internal or external resorption, or root canal therapy were not taken into account. Teeth were examined using a dental microscope (OMS2350; ZUMAX, Jiangsu, China) before being used. The external surface of the extracted teeth and the three-dimensional location of the apical foramen were examined using an endodontic microscope. To visualize any potential carious lesions, fissures, fractures, or resorption flaws of the root, an investigation was conducted. It took a 14x magnification to see these minute features. Teeth with root curvatures between 0° and 10° were selected for this study. An ultrasonic scaler was used to remove any leftover soft tissue or calculus after the external tooth surfaces had been properly cleansed with running water. Following a three-hour immersion in 2.5% sodium hypochlorite (NaOCL) to clean the tooth surfaces, they were stored in saline solution until instrumentation. To achieve a consistent root length of 12 mm, the teeth were decoronated using a diamond disc at the cementoenamel junctions. Allocation was concealed by using opaque sealed envelopes with sequential numbers (1–30), and the random sequence was generated using a website that generates random sequences. Using the website random.org, a straightforward randomization procedure was carried out, generating a random list for three groups. The material assignment was not concealed from the assistants or the primary operator. The assessing operator, however, was blinded. The inclusion criteria of the tooth were those teeth with whole root development, intact root surface, no indications of resorptions either internal or external, round root canal, curvature degree less than 10, and the root canal's dimensions close to the apical foramen allowed the insertion of file ISO15. However, the exclusion criteria of the tooth were teeth with more than one root canal, teeth with cracks, teeth with resorptions or caries, or previous root canal treatment.

### Root canal preparation and obturation

#### Root canal instrumentation

Using a #10 K-file (Dentsply-Maillefer, Ballaigues, Switzerland) until it was visible at the apical foramen and deducting 0.5 mm from the total measured length, the working length was visually determined. In accordance with the manufacturer's instructions, root canal instrumentation was carried out crown-down utilizing EdgeTaper Platinum rotary files up to file F4 (EdgeEndo, New Mexico, USA).

Following the use of each endodontic file, a 5 mL syringe and 30-gauge side-vented needle (Cerkamed, StalowaWola, Poland) were used to irrigate the root canals with 1 mL of 5.25% sodium hypochlorite (NaOCl) (Clorax, Oakland, California, USA). A final rinse with distilled water and paper points was used after the final irrigation, which included 3 mL of 5.25% NaOCl, distilled water, and 1 mL of 17% EDTA for one minute (MD Cleanser, Meta Biomed, Cheongwon-gun, Korea). The single cone technique was used to fill all of the roots.

### Experimental regimen

All roots were divided into three equal experimental groups

**Group I (control) :** roots were filled with gutta-percha cones (Dentsply Maillefer, Ballaigues, Switzerland) and the AH Plus sealer.

**Group II :** roots were filled with gutta-percha cones and the AH Plus sealer loaded with silver gold nanoparticles with ratio 1:1.

**Group III :** The canals were pretreated with two coats of NanoCare that were applied to the dentin surface of the root canal using micro brush. Every coat was left for 3 minutes for the natural evaporation of the solvent then filled with gutta-percha cones and AH Plus sealer [9].

Table 1 provided information about the materials utilized in this study, including their description, composition, manufacturing process, and lot number. For all roots, a final radiograph was taken to confirm the obturation and ensure adequate compaction. The access cavity was sealed with Cavit 482 © 2021 preliminary filling (3 M ESPE; St. Paul, MN, USA); samples were then stored for a week at 37 °C and 100% relative humidity to verify full setting.

**Table 1:** Tested sealer and its composition.

Endodontic Sealer	Specification	Composition	Manufacturer	Lot No
AH Plus	resin -based endodontic sealer	Paste A: Paste A: epoxy resins, calcium tungstate, zirconium oxide, silica, iron oxide pigments. Paste B: amines, calcium tungstate, zirconium oxide, silica, silicone oil, Urethane dimethacrylate (UDMA) resin	Dentsply, DeTrey GmbH, Konstanz, Germany	2202000328
Nano Care	Silver Gold Naoparticles	−99% Silver nanoparticles (AgNPs) with average size of 29.07 nm. −1%Gold nanoparticles (AuNPs) with average size 136.7 nm. −70% isopropyl alcohol.	Dental Nanotechnology, Katowice, Poland	270213

### Incorporation of Nano Care into AH Plus sealer

To find the screening concentrations of Nano Care Gold that will be added to AH Plus in order to achieve the lowest inhibitory concentration with the strongest antibacterial activity, a pilot study was conducted in the prior study [19]. 50%, 75%, and 100% of the evaluated concentrations of Nano Care Gold were used to modify the resin sealer. These concentrations were standardized by calculating that they were equal to one gram of the endodontic sealer (base and catalyst), which was weighed separately on an analytical scale (Adam Equipment Co. Ltd., MK10 0BD. UK) with a 10-4 g precision. Prior to applying the filling material or bonding system, the manufacturer advises using five to eight drops [20]. 1 drop is equivalent to about 15 ul of NG. Eight drops (120 uL, 100% concentration), six drops (90 uL, 75% concentration), and four drops (60 uL, 50% concentration) are so represented. Using micropipite, uL of each concentration was added to a container and allowed to dry (evaporation of the liquid carrier, isopropanol, based on manufacturer data). The powdered AgAu NP was combined with the endodontic sealer's base paste. Following the manufacturer's directions, the mixture was then mechanically mixed into the catalyst paste. The pilot study's findings showed that the concentration of 100% had the biggest zone of inhibition against E. faecalis growth, whereas 50% had the least [19]. In order to create a uniform mixture that was prepared for testing, 120 uL of nano gold was allowed to dry before being combined with the AH Plus sealer in a 1:1 ratio using a plastic spatula. To ensure that the silver gold nanoparticles were properly dispersed, the Nano Care Gold bottle was shaken each time before use [20].

### Mounting teeth specimens in acrylic blocks

Each root was positioned independently in blocks of polymethyl-methacrylate (PMMA) resin, vertically along its long axis. Specially constructed cylindrical Teflon molds were machine-milled to create the acrylic blocks. Each mold has an internal diameter of 20 mm and a height of 20 mm to accommodate the acrylic resin. A cover is attached to the top of this mold using two little pins. There is a rounded hole in the middle of this cover that is 5.0 mm in diameter, which can be used to find the center of the acrylic block. After setting the Teflon mold on the surveyor's base, a soft mixture of polymethyl-methacrylate (PMMA) resin was poured into it. The center of the acrylic block was measured using the mold cover. Then, while the mixture was still soft, the mold cover was taken off. A paralleling device (Surveyor) was used to mount each root inside the acrylic blocks, ensuring the specimens' centralization and alignment. Until the acrylic resin hardened, the root was left in situ. The acrylic block was removed from the mold by pressing its base with the finger.

### Push-out test

**Fig. 1:**
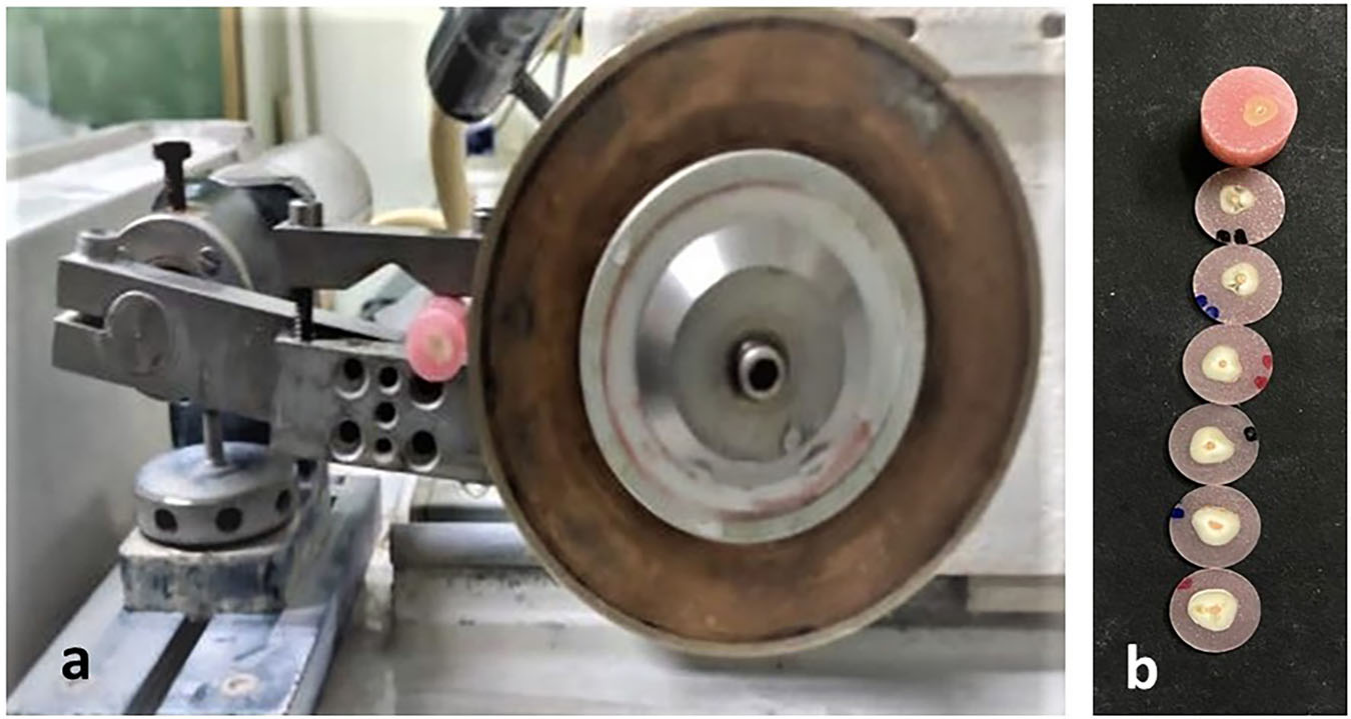
Preparation of specimen slide with microtome. **a**, **b** Preaparation of specimens slabs with microtome: where (**a**) represent specimen adjustment in the microtome, and (**b**) represents the obtained sliced specimens.

The acrylic blocks from each group were sectioned horizontally using a water-cooled diamond saw set at a moderate speed (Buehler Isomet 2000, Lake Bluff, IL, USA) to produce slices for the apical, middle, and coronal root areas that were 1 mm thick (Fig. 1a, b). A range of cylinder-shaped stainless-steel plunger diameters—1.0, 0.76, 0.50, and 0.35 mm—were used to load the filling material because they provided the longest coverage throughout the filling material without making contact with the canal wall. Three different pluggers were used as the root were sectioned into coronal, middle and apical sections thus the corresponding size were used according to the diameter of the section used and the data obtained were presented by their statistical means of different section accordingly to be analyzed.

**Fig. 2:**
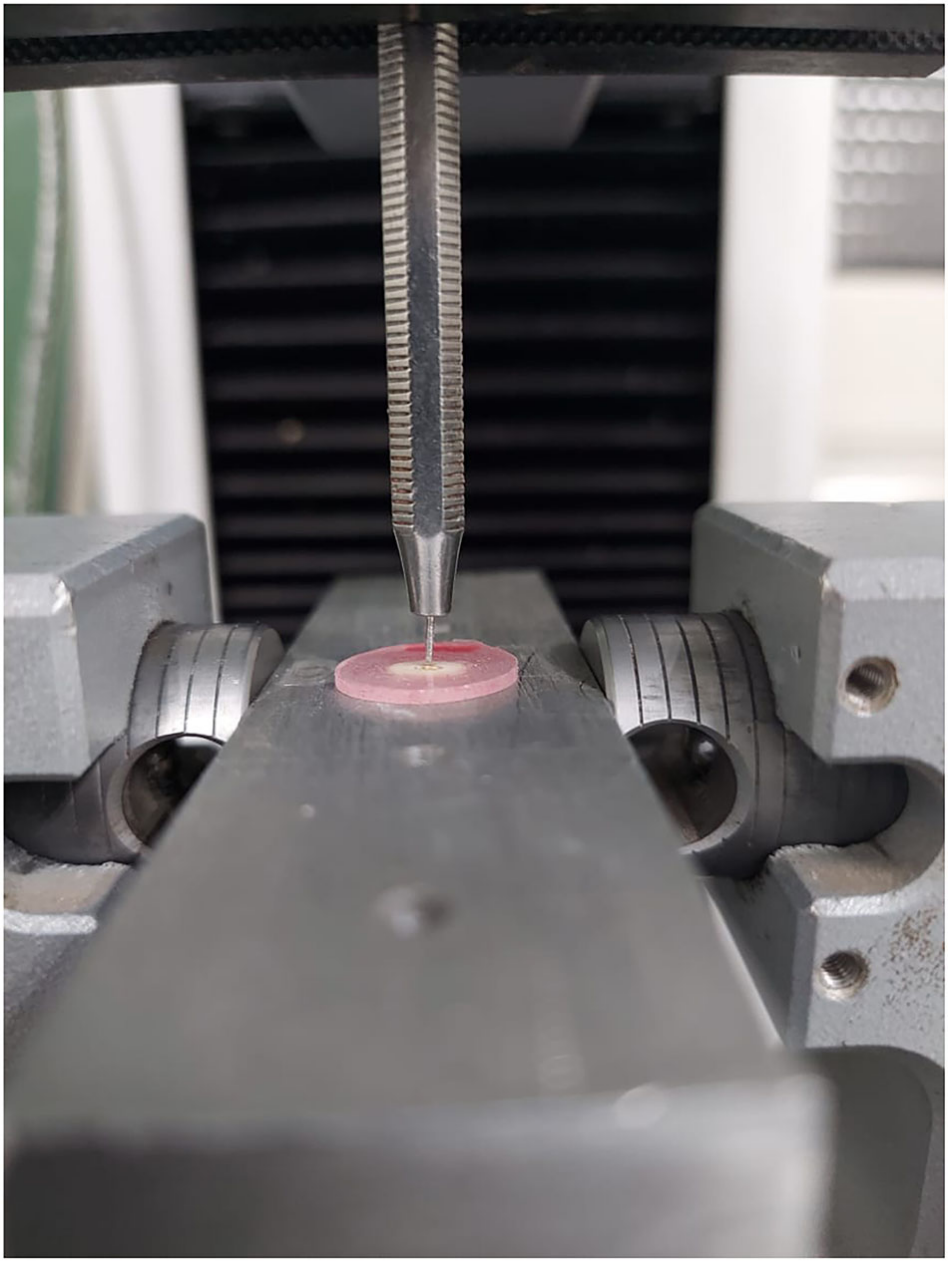
Push out testing.

A plunger was installed on the upper part of the Instron universal testing machine type 3345 England. To collect the data, Bluehill 3 version 3.3 software was used. Each slice was marked on its coronal side using an indelible marker because the samples were oriented apically to the coronal direction over a support jig to avoid any constriction interference. The test used a 500N load cell that was fastened to a loading fixture and run at a cross head speed of 1 mm/min (Fig. 2). The push-out bond strength was discovered to be the greatest value ever noted. The maximum failure load was measured in N and then converted to MPa. Using the following formula, the bond strength was determined by dividing the recorded peak load by the computed surface area: Adhesion area (mm^2^) / Maximum load (N) equals push-out bond strength (MPa). The adhesion area was calculated by using the following formula: ${{\rm{A}}}={{\rm{\pi }}}({{\rm{R}}}+{{\rm{r}}})[\left(\right.{{\rm{h}}}2+({{\rm{R}}}-{{\rm{r}}})2]0.5$. where π = 3.14, h is the slice's thickness, R is its coronal side radius, and r is its apical side radius. A digital caliper (Vonder, Curitiba, PR, Brazil) was used to measure the thickness of each slice, and a stereoscope (Leika MZ75, Meyer Instruments, Houston, TX, USA) and IM50 software (Leika IM50 Image management, Houston, TX, USA) were used to measure the coronal and apical radii. Each group's values were recorded, collated, and statistically examined [20, 21].

### Statistical analysis

The mean and standard deviation (SD) figures were used to display numerical data. To check for normalcy, Shapiro-Wilk's test was employed. Levene's test was used to determine whether the variances were homogeneous. The data was analyzed using a one-way ANOVA followed by Tukey's post hoc test for intergroup comparisons and a repeated measures ANOVA followed by Bonferroni post hoc test for intragroup comparisons. The data displayed a parametric distribution and variance homogeneity. Spearman's rank order correlation coefficient was used for correlation analysis. For all tests, the significance level was set at *p* < 0.05. R statistical analysis software, version 4.1.3 for Windows, was used to conduct the statistical analysis [22].

## Results

### Push-out bond strength

Table 2 displays the findings of comparisons between and within groups for push-out bond strength values. In terms of intergroup comparisons, there was a significant difference between groups I and II in both the coronal and middle areas (f = 5.26, *p* = 0.014 and f = 4.89, *p* = 0.018, respectively). Group (I) had the highest value, followed by group (II), and group (III) had the lowest value. Nonetheless, there was no significant difference between the groups in the apical portion (f = 0.85, *p* = 0.444). Group (II) had the greatest value (3.66 ± 0.72), followed by group (III) (2.90 ± 1.45), and group (I) had the lowest value (2.88 ± 1.73). In terms of intragroup comparisons, values recorded at various portions did not significantly differ in any group. All groups indicate non-significant differences in values recorded at various sections for intragroup comparisons (f = 1.39, *p* = 0.272 for group I; f = 2.29, *p* = 0.138 for group II; f = 3.00, *p* = 0.071 for group III).

**Table 2:** Inter and intragroup comparisons for push-out bond strength (MPa).

Section	Push-out bond strength (MPa) (Mean ± SD)			*f*-value	*p*-value
	Group (I)	Group (II)	Group (III)		
**Coronal**	2.53 ± 0.75^ABa^	2.78 ± 0.74^Aa^	1.82 ± 0.15^Ba^	5.26	0.014*
**Middle**	3.58 ± 1.18^ABa^	3.92 ± 1.63^Aa^	2.14 ± 0.58^Ba^	4.89	0.018*
**Apical**	2.88 ± 1.73^Aa^	3.66 ± 0.72^Aa^	2.90 ± 1.45^Aa^	0.85	0.444
**f-value**	1.39	2.29	3.00		
**p-value**	0.272	0.138	0.071		

Means with different upper and lowercase superscript letters within the same horizontal row and vertical column respectively are significantly different. *significant (*p* < 0.05).

## Discussion

Leakage around filling materials inside root canals is believed to be the main cause of the tooth's death cycle. As a result, maintaining the interfacial integrity of the intraradicular dentin and the obturation materials inside the root canals is crucial. Maintaining the sealing integrity of root canal fillings and avoiding bacterial infiltration from the oral cavity and periradicular tissues—which can result in problems after treatment—depend on how well root canal sealers adhere to dentin. Filling bond strength (FBS) can be utilized to gauge sealing capacity because of the strong inverse relationship between leakage and root filling bond strength [23]. The root canal filling materials' resistance to dislocation has been evaluated in vitro using the push-out test [24]. The advantage of this test is that the material can still generate a uniform shear strength when tested inside the root canal [25]. The push-out bond strength test is an indirect measure of sealing ability but a direct measure of bond strength. It is thought to be a helpful method for determining the materials' bonding strength and evaluating how effectively endodontic sealers attach to dentinal walls or core materials [26, 27]. Better bond strength, for instance, was associated with the substance's capacity to seal into dentinal tubules.

The most important practical applications of silver-gold nanoparticles are their antibacterial and antifungal characteristics. For teeth that have had endodontia, it can be considered a state-of-the-art antibacterial method. It has not yet been incorporated as an adjuvant into the root canal therapy regimen, despite its intriguing characteristics and possible advantages. Furthermore, it is yet unclear how they affect the adhesion and penetration of root canal sealers. The push-out bond strengths to the intraradicular dentin were evaluated in this study and compared between using the sealer by itself and using NanoCare Gold either as a pretreatment for canals or in combination with AH Plus sealer. Surprisingly, the preclinical trial's results showed that the push-out bond strength of utilizing the sealer alone and in combination with AgAu NPs did not differ significantly. The nanoparticles' size and shape properties may help to explain this. Spherical nanoparticles composed of nanocare gold typically have a diameter of 48 nm. Because they only have one point of contact with the substrate, spherical Ag-Au nanoparticles have a lower propensity to agglomerate, claim Lohbauer et al. [25]. Since methanol and isopropanol prevent the metal nanoparticles from aggregating, the manufacturer recommended using them as the liquid carrier to spread the particles. The mass of these nanoscale particles is also insignificant because of their unique characteristics, which include the predominance of electromagnetic forces between them that keep the particles in a state of random molecular motion and inhibit precipitation, sedimentation, and agglomeration. The Nanocare Gold bottle was thoroughly inspected prior to each usage in this trial to guarantee that the nanoparticles were consistently dispersed properly. Additionally, the variously shaped and sized gold and silver nanoparticles would function as an inorganic filler in the resin composite restorative materials, improving their physical characteristics [28].

The push-out bond strength results in the present investigation were substantially higher when the sealer was used alone or in combination with NanoCare Gold than when the NanoCare Gold was used as a canal pretreatment prior to the sealer application. As a proposed newly developed cavity disinfectant with potent antibacterial properties, NanoCare was chosen as a surface pretreatment material to be examined in this work [29]. Additionally, it seems that the bond strength of the AH Plus sealer was impacted by the first two coatings of NanoCare Gold that were applied to the root canals. This was probably caused by the sealer being applied inside dentinal tubules that EDTA had already opened. This stopped the sealer from fully entering and changed the way it interacted with the root dentin, which in turn changed how adherent the sealer was. AgNPs, which accounted for 99 percent of the particles in the solution, had an average size of 29.07 nm, according to Mackiewicz and Olczak-Kowalczyk's (2015) [15] microscopic study of NanoCare's chemical composition. AuNPs made up the remaining 1% of the particles, which had an average size of 136.7 nm[26]. These mingy amounts of gold attached to AgNPs may have created larger aggregates that clogged the dentinal tubules and prevented the sealer entry from reaching the maximum depth.

The current study's findings aligned with those of Borczyk and Pietranek (2009) [26], who discovered a coating of gold and silver nanoparticles at the tooth restoration interface as a result of pretreating the cavity walls with NanoCare Gold before applying a resin composite restoration. However, the results were challenged by Porenczuk et al. (2019) [16] and Ramasetty et al. (2018) [27], who found that while the cavity pretreatment with NanoCare Gold had no effect on the bond strength of resin composite restoration, it did cause a different failure mode after application. Other researchers also noticed and interpreted this association, which could be explained by the uniform layer of silver-gold nanoparticles that forms on the dentin surface following the evaporation of the liquid carrier in the formulation, which would increase the adhesive system's bonding area [9, 16, 30–32]. The discrepancy in the results may be because, in accordance with the manufacturer's instructions, nano silver gold should be applied to the dentin surface in five coats immediately following enamel etching and prior to primer and adhesive application using etch and rinse adhesive systems [14, 15] in order to prevent interfering with the bond strength. This is not the case in this study.

Group II exhibited the highest bond strength values throughout all coronal and middle portions, whilst the bond strength in apical third was not when compared to the other three tested groups. since the final root filling's result is significantly influenced by the sealer's flow rate. It seems that the flow of the sealer is unaffected by combining nanocare gold in a 1:1 ratio. It is possible for a sealer with a high flow rate to fill isthmuses, ancillary canals, and imperfections in canals. A root canal sealer should have a flow rate of at least 20 mm, per ISO 6876/2001 [33]. Particle size is one of the variables that affects the sealer's flow rate. The many spherical nanoparticles (round, discoid) that make up Nanocare Gold have a mean size of 48 nm and a single point of contact, which lowers the possibility of agglomeration and has no effect on the sealer's flow rate [25].

In every examined group, the coronal and intermediate parts' push-out bond strengths were noticeably greater than the apical regions'. This may be because the gutta-percha cone fits the coronal and middle portion of the root canal precisely, which improves the sealer's ability to penetrate the dentinal tubules. Anatomical and histological parameters, including the orientation of the dentine tubules, their number, which decreases apically, and their size, which may be impacted by dentine treatment, have been proposed to have an impact on the bonding of root dentin [34–38]. Yet, it would be beneficial to have the merits of sustained antibacterial properties of silver-gold nanoparticles at the apical portion were paint-on pooling might occurs. The null hypothesis postulated in this study concerning bond strength was rejected since significant differences in the bond strength were found among the tested experimental groups.

### Limitation of the study

A number of unavoidable restrictions, including as temperature, humidity, acidity, and the presence of various microbial species, may impact the clinical consideration of any laboratory investigation. All attempts were made, nevertheless, to reduce operator bias or variables. One operator carried out all of the clinical procedures in order to prevent operator impacts on the sealer's penetrating nature. In addition, this preclinical research is crucial to precisely identify and appropriately interpret the mechanism of action of this innovative endodontic treatment approach prior to clinical assessment.

### Recommendation

Although the physical and mechanical properties of the sealer have not yet been tested, the promising results of this group in this assessment method highlight the need for additional research into the new sealer formulation.

## Conclusions

While the usage of NanoCare Gold as a canal pretreatment prior to sealer application demonstrated their inferiority except for the root apical portion, experimental AH Plus sealer in conjunction with silver-gold nanoparticles obtained the most effective adhesion among the tested groups that were evaluated. Endodontic therapy may benefit greatly from the use of resin-based sealers in conjunction with silver-gold nanoparticles since this study may provide pertinent information that improves the sealers' clinical effectiveness. Moreover, it would be beneficial to have the merits of sustained antibacterial properties of silver-gold nanoparticles at the apical portion were paint-on pooling might occurs.

## Data Availability

The datasets used and/or analyzed during the current study are available from the corresponding author on reasonable request.
